# Ag(111) Remains Significantly Reduced In Situ under Simulated Ethylene Epoxidation Conditions

**DOI:** 10.1021/acs.jpclett.6c00522

**Published:** 2026-03-31

**Authors:** Elizabeth E. Happel, Toghrul Azizli, Gloria A. Sulley, Avery S. Daniels, Cole Easton, Adrian Hunt, Phillip Christopher, Iradwikanari Waluyo, Matthew M. Montemore, E. Charles H. Sykes

**Affiliations:** † Department of Chemistry, 1810 Tufts University, Medford, Massachusetts 02155, United States; ‡ Department of Chemical and Biomolecular Engineering, 5783 Tulane University, New Orleans, Louisiana 70118, United States; § National Synchrotron Light Source II, 8099 Brookhaven National Laboratory, Upton, New York 11973, United States; ∥ Department of Chemical Engineering, University of California, Santa Barbara, California 93106, United States; ⊥ Department of Chemical and Biological Engineering, 1810 Tufts University, Medford, Massachusetts 02155, United States

## Abstract

Direct ethylene epoxidation is among the highest value processes in the chemical industry, yet the reaction mechanism remains debated. A central question is whether the unpromoted Ag catalyst is metallic or oxidized under reaction conditions, as this determines the active oxidant species. Using ambient pressure X-ray photoelectron spectroscopy at chemical potentials simulating industrial conditions, we find that under oxidizing environments, nucleophilic oxygen (∼80% surface coverage) and some carbonate impurities (∼20% coverage) form on Ag(111). Upon switching to an industrially relevant 5:2 ethylene-to-oxygen ratio at 433 K, nucleophilic oxygen is consumed, leaving mostly surface carbonate and bare Ag. The Ag(111) surface maintains ∼50% exposed metallic sites under these conditions. This indicates that proposed mechanisms involving a fully oxidized surface may not represent the state of the surface under relevant reaction conditions and that bare Ag sites, which are necessary to form the oxametallacycle intermediate thought to drive selective epoxidation, are available.

## Graphical abstract

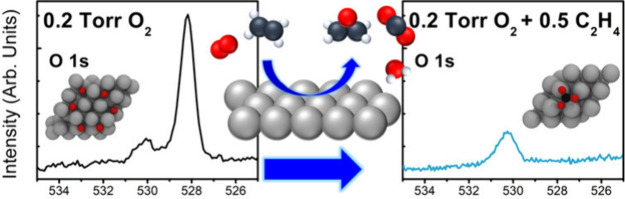

Ethylene oxide (EO) is an intermediate for the production of plastics, polyester, and various glycols, including ethylene glycol. The partial oxidation of ethylene by oxygen to form EO is among the highest-volume chemical processes in the industry, projected to reach approximately $43 billion/year by 2030.^1^ This reaction exemplifies a kinetically controlled reaction, where EO is the main product despite the much greater thermodynamic stability of the byproducts carbon dioxide and water.^2–4^ Silver-based catalysts are primarily employed for this reaction, achieving an EO selectivity of around 50% when unpromoted. Extensive academic and industrial research has improved this selectivity to 90% through the use of promoters such as Cl, Re, and Cs.^2,5,6^ Importantly, the nature of adsorbed oxygen on silver has been shown to substantially influence selectivity to EO.^7,8^

X-ray photoelectron spectroscopy (XPS) studies have identified two primary oxygen species on Ag surfaces: electrophilic (∼530.2 eV) and nucleophilic (∼528.4 eV).^8–14^ It is generally accepted that electrophilic oxygen predominates on Ag at lower temperatures and pressures, while nucleophilic oxygen forms at higher temperatures and pressures.^10,13,15–19^ Both species are widely believed to be composed of oxygen with different coordination sites that lead to differing selectivities for EO formation. For instance, Bukhtiyarov et al. examined Ag foils using temperature-programmed reaction (TPR) experiments with isotopically labeled oxygen and suggested that only electrophilic oxygen contributes to EO formation, while both electrophilic and nucleophilic oxygen generate CO_2_.^20^

However, other work has suggested that O_2_ is responsible for EO formation. For instance, some of the earliest studies by Campbell and co-workers indicated that low-coverage molecular O_2_ is involved in the rate limiting step for EO formation on a Ag(110) surface.^2,21^ Later investigations instead suggested that more fully oxidized Ag catalysts are essential for EO production.^22,23^ In contrast, studies by Linic and Barteau showed evidence for an oxametallacycle (OMC) intermediate that can form EO in which ethylene is bound to bare Ag and an O atom, indicating that the OMC formation requires both O and bare Ag sites not present on a fully oxidized surface.^24–26^

Given these open questions, in situ single-crystal and Ag foil studies of the ethylene epoxidation mechanism have garnered increased interest. Guo et al. utilized ambient pressure X-ray photoelectron spectroscopy (AP-XPS) to characterize oxygen species present at a maximum total pressure of 0.75 Torr of ethylene and oxygen, identifying several distinct oxygen species which were assigned as surface lattice oxygen, a dioxygen form of oxygen, and subsurface oxygen species.^27^ Most recently, investigations by Wachs, Flaherty, and others employing in situ Raman and XPS in conjunction with DFT have suggested that dioxygen oxygen or partially negative O_2_-like species may be the active species for ethylene epoxidation.^28–31^

Nevertheless, the precise chemical state of the surface under reaction conditions and the extent of surface oxidation remain open questions. Single-crystal studies offer the ability to more easily quantify the type and amount of surface oxygen than supported catalysts, but the difficulty of oxidizing Ag(111), which has a dissociative sticking probability of ∼5 × 10^–6^, requires oxidants like NO_2_ or high-pressure cells that can introduce contaminants. Furthermore, the majority of studies have focused on oxidizing conditions.^32^ However, ethylene is a reductant, and given that ethylene epoxidation is industrially performed in an ethylene-rich environment with an ethylene:oxygen ratio of ∼5:2, it is important to study the state of the Ag surface under this balance of oxidizing and reducing reactants.^33–35^ Herein, we demonstrate that at temperatures and pressures that simulate industrial conditions, ∼80% of the Ag surface is oxidized under 0.2 Torr oxygen and 433 K, whereas when ethylene is introduced at a 5:2 ratio, the surface becomes reduced and is composed of bare Ag sites and surface-bound carbonate.

While AP-XPS instruments cannot reach industrial pressures, one can study the adsorbates at the same chemical potential as Ag nanoparticle catalysts by working at a lower temperature (i.e., 433 K) that should yield equivalent surface coverages of the adsorbates at steady state (Table S2). Here, we use 433 K to match the surface chemical potential of O/Ag at simulated reactor conditions (523 K, 75 Torr O_2_). Therefore, to characterize Ag(111) in a purely oxidizing environment, we first saturate a roughened Ag(111) crystal with oxygen at 0.2 Torr and 433 K. To account for the effect of steps and defects, like those known to be important to support the formation of nucleophilic oxygen, the surface was lightly Ar^+^ sputtered before introducing reactant gases.^36^ After equilibration (∼90 min), the primary species observed was nucleophilic oxygen, with an XPS binding energy at around 528.4 eV (Figure 1A). This species is often associated with the p(4 × 4) reconstruction on Ag(111), and this type of nucleophilic oxygen is generally thought to favor the total combustion of ethylene.^13,18,19^ Additionally, a higher binding energy feature at around 530.2 eV was detected, which can be attributed to either electrophilic oxygen or carbonate impurities.^10,15–17^ Given the significant overlap in the binding energies of electrophilic oxygen and carbonate oxygen, we used C 1s spectra to quantify the amount of oxygen present as carbonate vs surface oxygen. The ∼288.1 eV peak in the C 1s spectra is associated with carbonate, and given the 3:1 ratio of oxygen to carbon in carbonate, our analysis (see S.I.) indicates that the dominant oxygen species constituting the 530.2 eV XPS feature is carbonate. Thus, under these conditions, assuming surface oxygen saturation as 0.375 monolayer (ML), in agreement with the saturation coverage of the p(4 × 4) reconstruction on Ag(111), ∼17% of the surface is covered by carbonate and ∼77% by nucleophilic oxygen, while ∼6% of the surface remains bare under oxidizing conditions. While there was not a deliberate addition of a carbon source to the gas environment, it is known that AP-XPS chambers in user facilities typically have a residual background pressure of carbon-containing contaminants, which, when oxygen is present, can lead to the buildup of carbonate on otherwise clean surfaces.^37–39^

**Figure 1:**
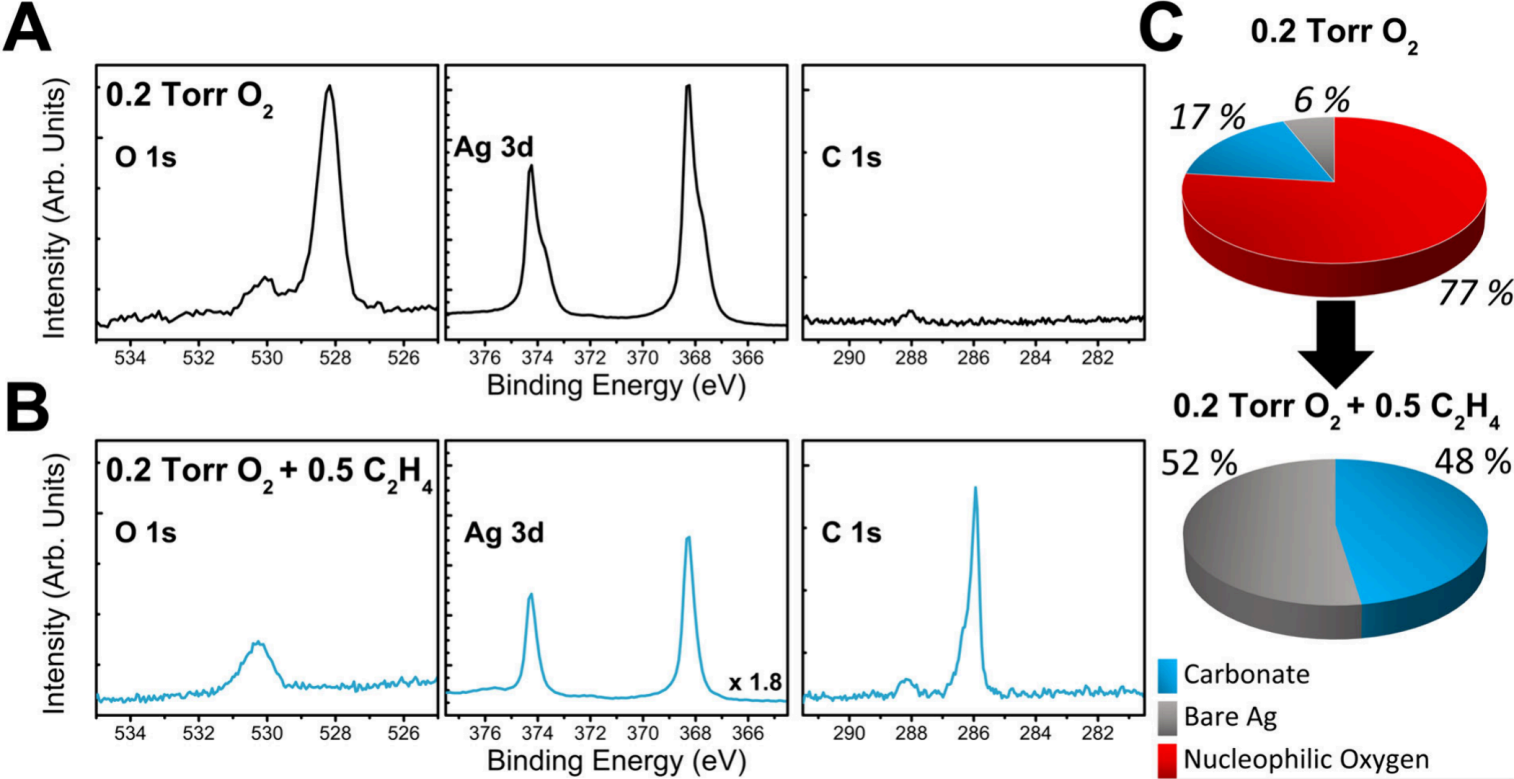
**AP-XPS spectra showing surface species on Ag(111) in oxidizing vs epoxidation conditions**. O 1s, Ag 3d and C 1s spectra of Ag(111) under (A) 0.2 Torr O_2_ at 433 K and (B) 0.2 Torr O_2_ and 0.5 Torr C_2_H_4_ at 433 K. The C 1s and Ag 3d spectra were taken at the photon energy 500 eV and O 1s at 760 eV (Ag 3d were also corrected for the lower electron inelastic mean free path (IMFP) at higher background pressures). (C) Surface composition under 0.2 Torr oxygen and 0.2 Torr oxygen + 0.5 Torr ethylene.

Upon the introduction of 0.5 Torr of ethylene to the existing 0.2 Torr of oxygen at 433 K, which is equivalent to 0.1 bar of oxygen at 523 K, a distinct gas-phase ethylene doublet feature appears at ∼286 eV in the C 1s spectra (Figure 1B and 2B) and the coverage of nucleophilic oxygen decreases to zero as seen in Figure 1B and 2A. It is also apparent from these XPS spectra that the total oxygen coverage decreases to ∼50%, and by quantifying the oxygen-to-carbon ratios we find that all of this oxygen is associated with carbonate species, appearing at a binding energy of ∼530.4 eV.^10,40^ Notably, upon ethylene introduction, the carbonate increases from covering ∼20% of the surface to ∼50%, in agreement with previous reports of carbonate formation under ethylene epoxidation conditions, which has been suggested to be a spectator in the reaction.^37,39–41^

**Figure 2:**
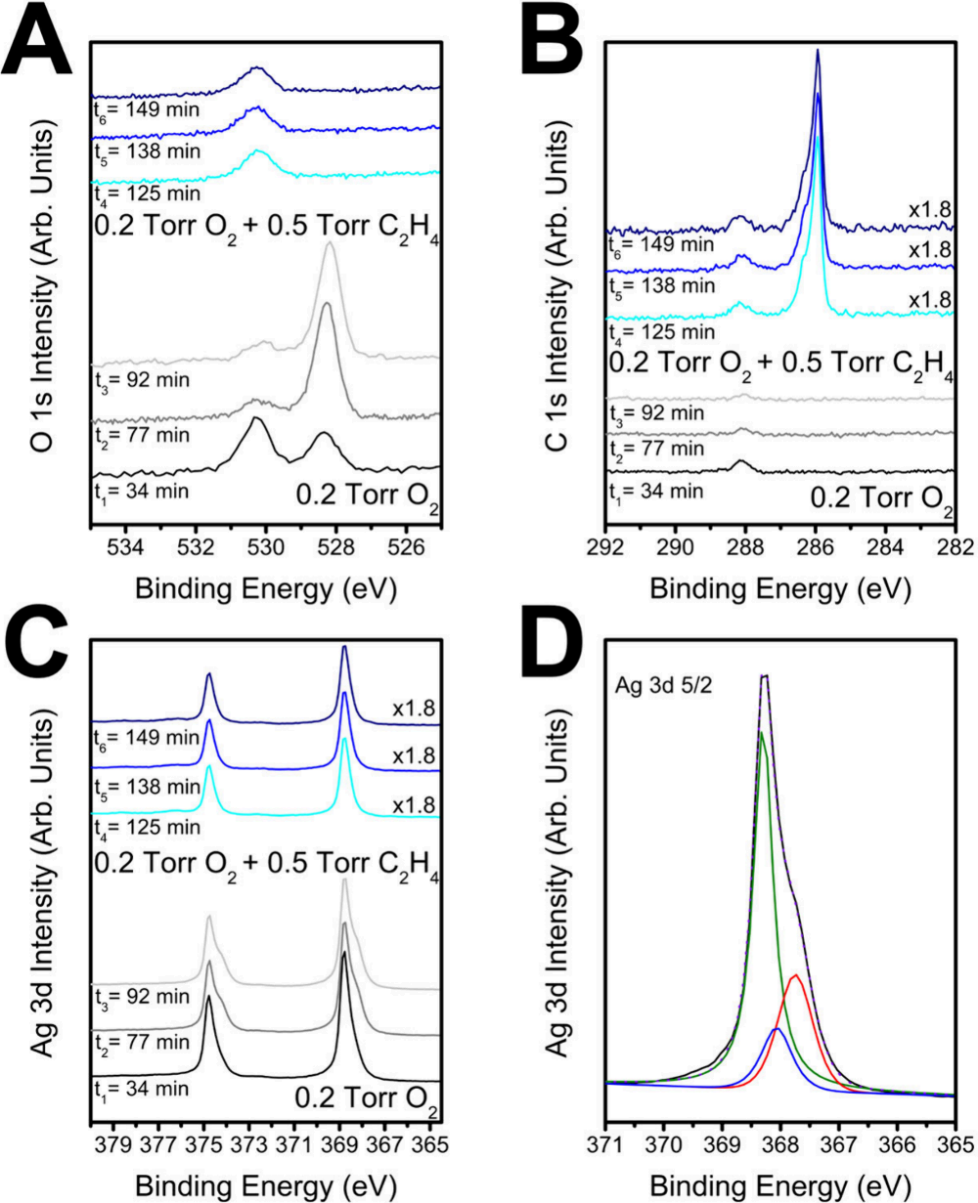
**Equilibration of surface adsorbates on Ag(111) upon changing from purely oxidizing to simulated ethylene epoxidation conditions**. The equilibration of O 1s, C 1s, and Ag 3d spectra over time is shown in (A), (B), and (C), respectively, after initial equilibration of Ag(111) under 0.2 Torr oxygen and then with the simultaneous introduction of 0.5 Torr of ethylene to the environment at 433 K. (D) Fitted Ag 3d_5/2_ spectra after 92 min in 0.2 Torr oxygen at 433 K with a distinct shoulder at higher binding energy (red) assigned to an oxygen-induced Ag reconstruction and Ag in furrows below oxygen (blue).^42^ Where noted, XPS peak intensity was corrected for the lower inelastic mean free path at higher chamber pressure.

Further investigation of the surface species present under simulated epoxidation reaction conditions was conducted by measuring the Ag 3d spectra. Under purely oxidizing conditions (0.2 Torr oxygen), a lower binding energy shoulder at ∼367.8 eV in the Ag 3d spectra was observed (Figure 2). While the oxidation of most metals results in a shift of the metal d orbitals to higher binding energies, oxide formation on Ag surfaces (i.e., Ag_2_O) results in a unique shift to lower BEs (∼367.3 eV) due to final-state effects.^3,42–44^ The binding energy of the observed Ag 3d shoulder in this work at ∼367.8 eV corresponds to the formation of a reconstructed surface oxide layer, such as the p(4 × 4) structure that is often associated with nucleophilic oxygen on Ag(111).^42,45^ Thus, under these purely oxidizing conditions at 433 K, the Ag surface is reconstructed, but is not fully oxidized.

It is clear from Figure 2 that when ethylene is introduced to Ag(111) under 0.2 Torr oxygen, the nucleophilic oxygen is rapidly consumed and the Ag 3d spectra return to the same shape and BE as metallic Ag(111) with some carbonate (Figure S1). Due to our use of lower sample temperatures (chosen to match the chemical potential of an industrial reactor system which operates ∼523 K) we expect our reaction rates to be slower.^46^ However, all the rates should decrease with temperature; therefore, we allowed enough time for each surface species to reach a steady state under simulated reaction conditions. The time required to reach steady state, at which no changes were observed in the O 1s, C 1s or Ag 3d spectra coverages, was ∼1.5 h under 0.2 Torr oxygen and ∼2.5 h under 0.2 Torr oxygen and 0.5 Torr ethylene (Figure S3). Specifically, low coverages of carbonate can lead to a small shoulder in Ag 3d spectra, offset by only ∼0.2 eV from the metallic peak.^39,40,47^ The simultaneous loss of both nucleophilic oxygen species in the O 1s spectra and the reconstructed Ag shoulder of the Ag 3d spectra further confirms the assignment of this shoulder to a surface reconstruction like the p(4 × 4) associated with nucleophilic oxygen. Both shoulders associated with carbonate and nucleophilic oxygen always remain distinct from bulk oxide formation. Quantification of the carbonate coverage reveals that under simulated reaction conditions with the industrially relevant 5:2 ethylene to oxygen ratio, the Ag surface is ∼50% metallic with ∼50% covered by carbonate. This suggests that under reaction conditions there are bare Ag sites available and therefore intermediates which require metallic Ag like the OMC could form.

To account for moderate variations in reactor conditions, as well as possible differences in the exact surface chemical potential between our model studies and EO reaction conditions, we varied the surface temperature both above and below 433 K. Specifically, we performed experiments at 418 K (calculated to be +0.04 eV in O_2_ chemical potential, relative to 433 K) and 463 K (−0.08 eV in O_2_ chemical potential relative to 433 K). We note that while we match surface chemical potentials of oxygen on Ag(111) of our model system to reactor conditions, our thermodynamic modeling, which interpolates between experimental entropies and enthalpies, is expected to be more accurate than these variations. In these experiments, while changes in temperature affect the distribution of oxygen species, the Ag consistently remains reduced (Table 1). We also note that even under these higher temperature conditions, we do not see the formation of any new oxygen species, including subsurface oxygen which would be expected at ∼531 eV, likely due to the high ratio of ethylene to oxygen that leads to a depletion of surface oxygen as observed in our XPS data (Figure 1B).^48,49^ Both higher and lower temperatures result in decreased carbonate coverages. In fact, at temperatures above 460 K carbonate will begin to decompose.^50^ Therefore, even at the highest carbonate coverages, the total oxygen coverage remains low, and the Ag 3d spectra consistently indicate a metallic state.

**Table 1:** Effect of Temperature on Surface Coverage under Reaction Conditions

	*Coverage, ML*			
	**Electrophilic Oxygen**	**Nucleophilic Oxygen**	**Carbonate**	**Bare Ag**
**0.2 Torr O**_**2**_ **+ 0.5 Torr C**_**2**_ **H**_**4**_ **418 K**	0.0 ± 0.05	0.0 ± 0.05	0.2 ± 0.05	0.7 ± 0.05
**0.2 Torr O**_**2**_ **+ 0.5 Torr C**_**2**_ **H**_**4**_ **433 K**	0.0 ± 0.05	0.0 ± 0.05	0.5 ± 0.05	0.5 ± 0.05
**0.2 Torr O**_**2**_ **+ 0.5 Torr C**_**2**_ **H**_**4**_ **463 K**	0.0 ± 0.05	0.0 ± 0.05	0.3 ± 0.05	0.6 ± 0.05

To test whether DFT-calculated energetics are consistent with a metallic, carbonate-covered Ag surface being more stable than an oxidized surface under simulated reaction conditions, we constructed a phase diagram using ab initio atomistic thermodynamics as shown in Figure 3. Our results are consistent with previous studies comparing the p(4 × 4) oxygen reconstruction to adsorbed O and bare Ag.^51–53^ Furthermore, our DFT predictions are consistent with the experimental results in that metallic Ag with carbonate is more stable than the p(4 × 4) oxygen reconstruction under these experimental conditions (see Figure 3), providing further support of the experiments that demonstrate that around half of the Ag surface remains metallic under simulated reaction conditions. Thus, the presence of bare Ag sites and carbonate species should be considered in future models aimed at understanding the mechanism of ethylene epoxidation.

**Figure 3:**
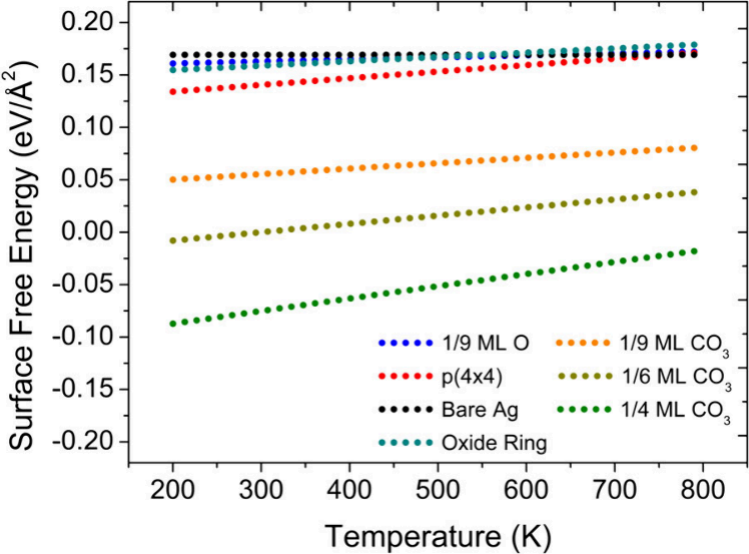
**DFT-calculated phase diagram showing the stability of a metallic and carbonate-covered Ag surface under simulated reaction conditions**. Surface free energies for bare Ag(111), O/Ag(111) at 1/9 ML, the p(4 × 4) reconstruction, and CO_3_/Ag­(111) at three different coverages. Conditions: *p*O_2_ = 0.2 Torr, *p*C_2_H_4_ = 0.5 Torr, *p*H_2_O = 0.01 Torr.

This work demonstrates that under purely oxidizing conditions and at a chemical potential that is equivalent to that of the oxygen partial pressure and temperature of simulated industrial EO synthesis conditions, nucleophilic oxygen is the dominant species on the surface of Ag(111). This is consistent with an oxygen-induced Ag reconstruction, such as the p(4 × 4) structure. This nucleophilic oxygen coverage constitutes approximately 80% of a monolayer, leaving a small portion of exposed metallic Ag and carbonate formed from the chamber background. In contrast, upon the introduction of ethylene at the industrial ethylene:oxygen 5:2 ratio, nucleophilic oxygen is rapidly consumed, resulting in a metallic Ag surface with only carbonate present that occupies approximately 50% of the Ag(111) surface. Together, these results indicate that at industrially relevant reactant ratios, ethylene acts as a powerful reductant, quickly consuming nucleophilic oxygen and leaving only carbonate on the otherwise bare metallic Ag surface. This means that mechanistic models of ethylene epoxidation on Ag should not assume the surface is fully oxidized; rather, metallic Ag sites and carbonate appear to dominate the surface. Future studies are aimed at exploring the effect of common ethylene epoxidation promotors on the state of Ag.

## Supplementary Material



## Notes

Detailed experimental and computational methods, XPS analysis details, supporting XPS spectra and system phase diagram (PDF)
