# Supplementary material for: Ag(111) Remains Significantly Reduced In Situ under Simulated Ethylene Epoxidation Conditions
Source: J Phys Chem Lett. 2026 Mar 31;17(14):4229–34. doi: 10.1021/acs.jpclett.6c00522 (PMC13071909; doi:10.1021/acs.jpclett.6c00522)
Supplement: Supplementary file 1 [file jz6c00522_si_001.pdf]

Supplementary Information:

Ag(111) Remains Significantly Reduced In Situ Under Simulated Ethylene Epoxidation Conditions

Elizabeth E. Happel[1], Toghrul Azizli[2], Gloria A Sulley[2], Avery S. Daniels[1], Cole Easton[1], Adrian Hunt[3], Phillip Christopher[4], Iradwikanari Waluyo[3,*], Matthew M. Montemore[2,*] and E. Charles H. Sykes[1,5*]

[1]Department of Chemistry, Tufts University, Medford, MA 02155, United States.

[2]Department of Chemical and Biomolecular Engineering, Tulane University, New Orleans, Louisiana 70118, United States.

[3]National Synchrotron Light Source II, Brookhaven National Laboratory, Upton, NY 11973, United States.

[4]Department of Chemical Engineering, University of California, Santa Barbara, CA 93106, United States

[5]Department of Chemical and Biological Engineering, Tufts University, Medford, MA 02155, United States.

Corresponding Author Information: iwaluyo@bnl.gov, mmontemore@tulane.edu, charles.sykes@tufts.edu

Experimental Methods

Ambient pressure X-ray photoelectron spectroscopy (AP-XPS) data were collected at the In situ and Operando Soft X-ray spectroscopy beamline (IOS, 23-ID-2) of the National Synchrotron Light Source II (NSLS-II) at Brookhaven National Laboratory.[1] The Ag(111) sample was mounted on a pyrolytic boron nitride (PBN) heater and temperature was measured with a K type thermocouple located between the back of the sample and heater. The crystal was cleaned by repeating cycles of Ar^+ sputtering and annealing to 900 K until XPS spectra showed no impurities. High purity O_2 (Matheson, 99.994%) and C_2H_4 (Matheson, 99.95%) were introduced to the chamber using precision leak valves. Photon energies of 760 and 500 eV were used to generate photoelectrons with the same kinetic energies of ~200 eV for O 1s and C 1s spectra respectively. Reference Ag 3d spectra were taken at both photon energies and, in addition to the Fermi edge, were used to calibrate binding energy shifts. The XPS spectra were analyzed with CasaXPS, linear backgrounds were used, and peaks fit as described in detail in Table S1.

Computational Methods

DFT Calculations

DFT calculations were performed using the Vienna Ab initio Simulation Package (VASP).[2,3] For the exchange-correlation potential, the Perdew−Burke−Ernzerhof (GGA-PBE)[4] functional was used, coupled with Tkatchenko-Scheffler (TS) dispersion corrections.[5] The projector-augmented wave method was utilized to treat the electron-ion interactions, and the plane wave cutoff energy was 400 eV. The relaxation of each geometry was done with 10^{-5} eV electronic energy and 0.03 eV/Å force tolerances. The Table S1 below shows the cell sizes and the corresponding k points sampled. The bottom two layers of each cell were fixed at their bulk positions.

In our thermodynamic calculations, we included a few possible oxygen-silver structures to compare stability with the CO_3-covered surfaces: the well-known p(4x4) reconstruction[6], localized "oxide rings"[7] centered on an Ag vacancy, and 1/9 ML coverage of atomic O. These structures are well-described in previous work. Previous DFT studies have predicted that the p(4x4) reconstruction is the most stable structure under moderately oxidizing conditions and without any reductant, such that Ag(111) transitions from a clean surface to the p(4x4) reconstruction to a bulk oxide.[8] This is consistent with our predictions that the p(4x4) reconstruction is the most stable structure without any carbon.

Table S1. **Cell sized and corresponding k points sampled for calculated structures.**

Species	Surface Cell Sizes	K-point grid
Bare Silver	3 × 3 × 4	7 × 7 × 1
Oxide Ring	5 × 5 × 4	5 × 5 × 1
p-(4x4)	4 × 4 × 5	5 × 5 × 1
1/9 O ML	3 × 3 × 4	7 × 7 × 1
1/4 CO_3 ML	2 × 2 × 4	10 × 10 × 1
1/6 CO_3 ML	2 × 3 × 4	10 × 7 × 1
1/9 CO_3 ML	3 × 3 × 4	7 × 7 × 1

Vibrational Calculations

Harmonic vibrational energy calculations were carried out for the surfaces with 1/9 O ML, oxide ring, p-(4×4) reconstruction, and CO_3 with different coverages. Only adsorbates on the surface were displaced while all metal atoms were fixed. The calculations were performed with 0.005 Å displacements and 2×10^{-6} eV electronic energy criterion.

Phase Diagram

All thermodynamic parameters were computed using the thermochemistry module of the Atomic Simulation Environment (ASE)[9], with harmonic vibrational frequencies and DFT electronic energies as calculated input. The surface free energy as a function of temperature and partial pressures is given by:

$$\gamma(T) = \frac{1}{A_{surf}}\left(F_{system}(T) + 2N_c\mu_{H_2O}(T, P_{H_2O}) - \frac{N_O}{2}\mu_{O_2}(T, P_{O_2}) - \frac{N_C}{2}\mu_{C_2H_4}(T, P_{C_2H_4})\right)$$

where N_C and N_O denote the number of carbon and oxygen atoms, respectively, in the system, and A_{surf} is the surface area of the unit cell. F_{system} represents the Helmholtz free energy of the system, while μ_{H_2O}, μ_{O_2} and $\mu_{C_2H_4}$ correspond to the chemical potentials of gaseous water, oxygen, and ethylene, respectively.

The Helmholtz free $F_{system}(T)$ energy was computed within the harmonic approximation using ASE's HarmonicThermo class. For gaseous water and ethylene, chemical potentials were calculated in the ideal gas limit using ASE's IdealGasThermo class.

Due to the inaccuracy of DFT-GGA in describing the ground state energy of O_2, an alternative approach was employed to determine its bond strength and thus chemical potential.[10] The DFT energy of O_2 was derived indirectly via:

$$E_{O_2,gas} = 2 * (E_{H_2O,gas} - E_{O_2,gas} + \Delta ZPE + \Delta_f H^{\circ}_{0,H_2O})$$

where ΔZPE is the zero-point energy correction and $\Delta_f H^{\circ}_{0,H_2O}$ is the standard formation enthalpy of water at 0 K, both taken from tabulated values.[10] The chemical potential of O_2 was then computed as:

$$\mu_{O_2}(T, P_{O_2}) = E_{O_2,gas} + k_B T ln\left(\frac{P}{P^0}\right) + h_{int}(T) - h(0) - T\big(s_{int}(T) - s(0)\big)$$

Here, h_{int} and s_{int} are the molar enthalpy and entropy, respectively, interpolated from the NIST thermochemical tables for O_2.[11]

XPS Spectral Analysis

Table S2. Description of components of XPS spectra

C 1s Component	Binding Energy	FWHM	Line Shape (L:G)
Carbonate	288.0-288.2	0.5	50:50
Gas Phase Ethylene	285.9	0.3	70:30
Gas Phase Ethylene	286.3	0.4	70:30

O 1s Component	Binding Energy	FWHM	Line Shape (L:G)
Electrophilic	530.1-4	1	50:50
Nucleophilic	528.1-2	0.8	50:50

Ag 3d 5/2 Component	Binding Energy	FWHM	Line Shape (L:G)
Metallic Ag	368.2-3	0.4	100:0
Reconstructed Ag	368.7-8	0.7	50:50
Carbonate on Ag	368.0-1	0.6	70:30

All spectra were corrected with a relative sensitivity factor (RSF) for each photon energy used. Where necessary, peak intensity was corrected for the decreased electron inelastic mean free path (IMFP) resulting from increased chamber pressure. Monolayer coverages were calculated with respect to a monolayer of Ag(111). To do this the Ag $3d_{5/2}$ spectra were first corrected for spin orbit splitting and RSF as well as IMFP as the Ag 3d reference spectra were taken at photon energies more surface sensitive to C 1s and O 1s species. The fraction of the total Ag 3d intensity attributed to the surface layer of Ag, 0.23 and 0.35 at 760 and 500 eV, respectively, was calculated using the electron escape depth for each photon energy; and these values were used to extract a monolayer reference for coverage calculations.

Additional confirmation of equilibrium coverage was performed by measuring the Ag $3d_{5/2}$ spectra with 500 eV photon energy (KE ~150 eV). With this higher surface sensitivity, the apparent formation and saturation of a surface reconstruction like the p(4x4) results in the growth of a lower binding energy shoulder in the Ag 3d spectra (Fig 2). The ratio of the reconstructed Ag 3d shoulder to the total Ag monolayer results in an equilibrium coverage of ~0.7 of a complete monolayer (1:1 with Ag atoms at the surface). This coverage aligns well with the equilibrated coverage of nucleophilic oxygen measured in the O 1s spectra at ~0.288 ML (1:1 with Ag atoms at the surface).

Given the known saturation coverage of p(4x4) on Ag(111) as 0.375 ML, the fraction of the Ag surface that should be reconstructed by 0.288 ML of nucleophilic oxygen (i.e. 0.288/0.375) gives ~0.77 of saturated p(4x4) surface. Meaning, that a coverage of 0.288 ML of nucleophilic oxygen should correspond ~0.77 ML of reconstructed Ag given the structure of p(4x4); which is in good agreement with the measured Ag area above.[12–15] We note that the fractional oxygen and reconstructed Ag coverages of the surface align well, further confirming the surface coverage calibration. In order to present a more realistic surface coverage of oxygen species, a saturated coverage of the p(4x4) reconstruction (0.375 ML O atoms to surface Ag atoms on Ag(111)) was used as a reference for % monolayer calculations throughout the text.

Table S3. O_2 Free Energy Corrections Under Different Reaction Environments

T (K)	P (Torr)	P (Bar)	P (Pa)	Free Energy Correction to O_2 (eV)
433	1		133.3	-1.09
433	0.2		26.66	-1.15
513		0.1	10000	-1.12
523		0.1	10000	-1.15
523		1	100000	-1.04
570		1	100000	-1.15
523		10	1000000	-0.94
620		10	10000000	-1.15

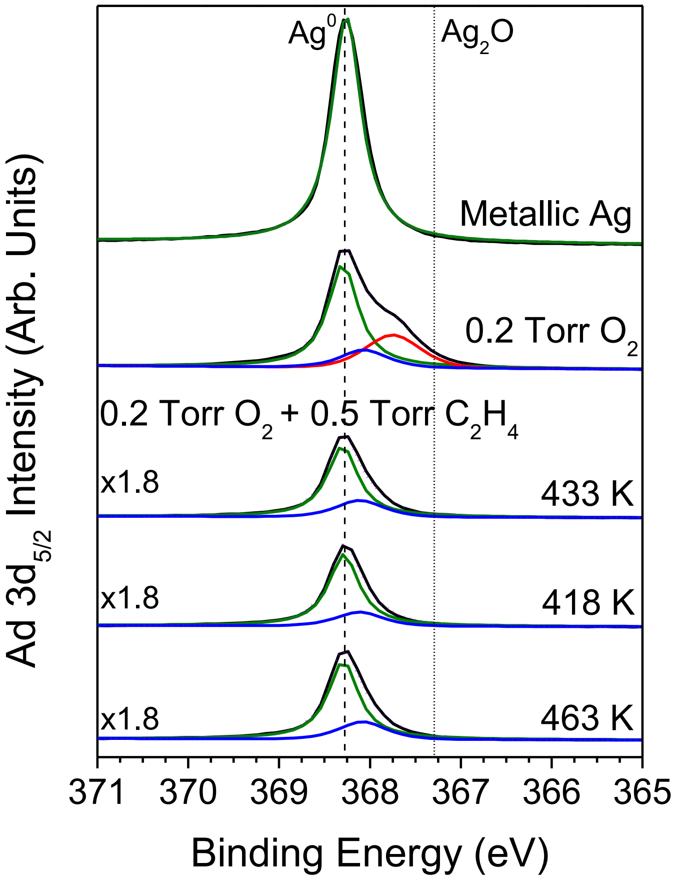

Figure S1. The formation and loss of a shoulder on Ag 3d$_{5/2}$ spectra of Ag(111) consistent with Ag restructuring under oxidizing and reducing conditions. There is a shift in the oxidation state of Ag from metallic (green trace) under UHV conditions, to equilibrated with a reconstructed atomic oxygen layer (red trace) under 0.2 Torr O_2, followed by a return to predominately metallic under more reducing conditions (0.2 Torr O_2 and 0.5 Torr C_2H_4). While the red trace is typically associated with an oxygen-induced surface reconstruction, it is not present under more reducing conditions. A small feature with a slight offset (~0.2 eV) from the metallic BE appears and remains stable in oxidizing and reducing conditions. This feature (blue trace) is often assigned to Ag in the furrows below O atoms or is associated with oxygen containing surface species present at higher coverages of e.g. carbonate.[12–14]

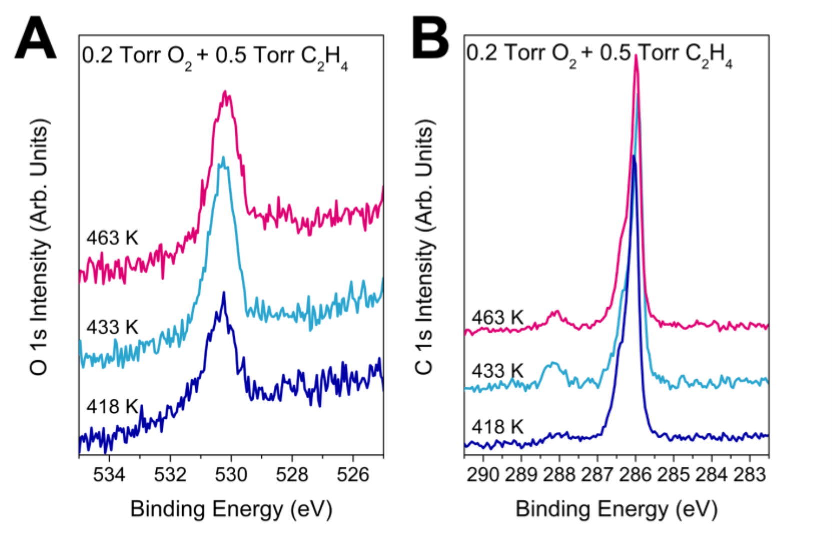

Figure S2. The effect of temperature on the surface coverage on Ag(111) under simulated ethylene epoxidation conditions. Increasing the temperature of a Ag(111) sample under 0.2 Torr O_2 and 0.5 Torr C_2H_4 from 418 K to 463 K reveals an increase in carbonate in the O 1s (A) and C 1s (B) spectra at intermediate (teal) temperatures and a reduction at higher (pink) and lower (dark blue) temperatures.

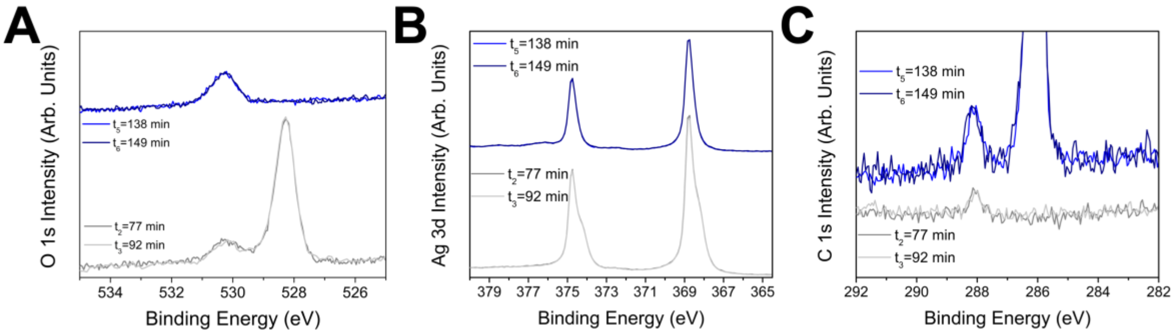

Figure S3. Equilibration surface species under oxidizing and reducing conditions. XPS spectra were continuously collected at 0.2 Torr O_2 at 433 K (grey) until no further changes in line shape were observed in any of the (A) O 1s, (B) Ag 3d, or (C) C 1s spectra before introducing 0.5 Torr C_2H_4 where XPS spectra (blue) continued to be collected again until no changes were observed.

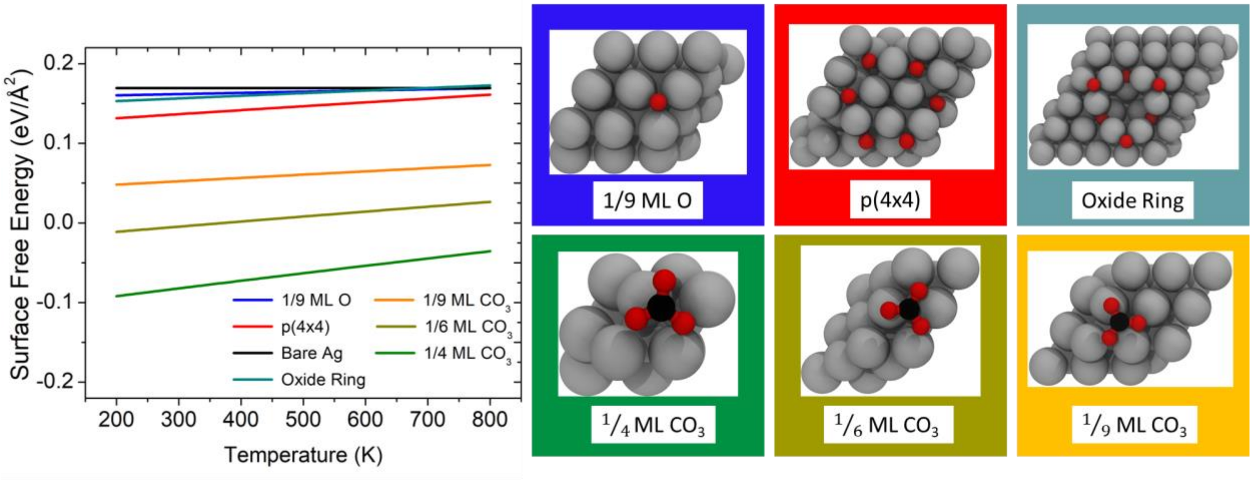

Figure S4. DFT-calculated phase diagram showing the stability of a metallic and carbonate-covered Ag surface under simulated reaction conditions. Surface free energies for bare Ag(111), O/Ag(111) at 1/9 ML, the p(4x4) reconstruction, and CO_3/Ag(111) at three different coverages. Conditions: pO_2 = 75 Torr, pC_2H_4 = 75 Torr, pH_2O = 7.5 Torr.

References

(1) Waluyo, I.; Hunt, A. Ambient Pressure X-Ray Photoelectron Spectroscopy at the IOS (23-ID-2) Beamline at the National Synchrotron Light Source II. *Synchrotron Radiat. News* **2022**, *35* (3), 31–38. https://doi.org/10.1080/08940886.2022.2082180.

(2) Kresse, G.; Furthmüller, J. Efficiency of Ab-Initio Total Energy Calculations for Metals and Semiconductors Using a Plane-Wave Basis Set. *Comput. Mater. Sci.* **1996**, *6* (1), 15–50. https://doi.org/10.1016/0927-0256(96)00008-0.

(3) Kresse, G.; Hafner, J. Ab Initio Molecular Dynamics for Liquid Metals. *Phys. Rev. B* **1993**, *47* (1), 558–561. https://doi.org/10.1103/PhysRevB.47.558.

(4) Perdew, J. P.; Burke, K.; Ernzerhof, M. Generalized Gradient Approximation Made Simple [Phys. Rev. Lett. 77, 3865 (1996)]. *Phys. Rev. Lett.* **1997**, *78* (7), 1396–1396. https://doi.org/10.1103/PhysRevLett.78.1396.

(5) Tkatchenko, A.; Scheffler, M. Accurate Molecular Van Der Waals Interactions from Ground-State Electron Density and Free-Atom Reference Data. *Phys. Rev. Lett.* **2009**, *102* (7), 073005. https://doi.org/10.1103/PhysRevLett.102.073005.

(6) Schmid, M.; Reicho, A.; Stierle, A.; Costina, I.; Klikovits, J.; Kostelnik, P.; Dubay, O.; Kresse, G.; Gustafson, J.; Lundgren, E.; Andersen, J. N.; Dosch, H.; Varga, P. Structure of Ag(111)-p(4x4)-O: No Silver Oxide. *Phys. Rev. Lett.* **2006**, *96* (14), 146102. https://doi.org/10.1103/PhysRevLett.96.146102.

(7) Andryushechkin, B. V.; Shevlyuga, V. M.; Pavlova, T. V.; Zhidomirov, G. M.; Eltsov, K. N. Adsorption of O2 on Ag(111): Evidence of Local Oxide Formation. *Phys. Rev. Lett.* **2016**, *117* (5), 056101. https://doi.org/10.1103/PhysRevLett.117.056101.

(8) *Thermodynamic and spectroscopic properties of oxygen on silver under an oxygen atmosphere - Physical Chemistry Chemical Physics (RSC Publishing) DOI:10.1039/C5CP00342C.* https://pubs.rsc.org/en/content/articlehtml/2015/cp/c5cp00342c (accessed 2026-01-21).

(9) Hjorth Larsen, A.; Jørgen Mortensen, J.; Blomqvist, J.; Castelli, I. E.; Christensen, R.; Dułak, M.; Friis, J.; Groves, M. N.; Hammer, B.; Hargus, C.; Hermes, E. D.; Jennings, P. C.; Bjerre Jensen, P.; Kermode, J.; Kitchin, J. R.; Leonhard Kolsbjerg, E.; Kubal, J.; Kaasbjerg, K.; Lysgaard, S.; Bergmann Maronsson, J.; Maxson, T.; Olsen, T.; Pastewka, L.; Peterson, A.; Rostgaard, C.; Schiøtz, J.; Schütt, O.; Strange, M.; Thygesen, K. S.; Vegge, T.; Vilhelmsen, L.; Walter, M.; Zeng, Z.; Jacobsen, K. W. The Atomic Simulation Environment—a Python Library for Working with Atoms. *J. Phys. Condens. Matter* **2017**, *29* (27), 273002. https://doi.org/10.1088/1361-648X/aa680e.

(10) *Chemisorbed Oxygen on the Au(321) Surface Alloyed with Silver: A First-Principles Investigation | The Journal of Physical Chemistry C.* https://pubs.acs.org/doi/10.1021/jp511884k (accessed 2025-08-12).

(11) NIST Office of Data and. *NIST Chemistry WebBook, O_2.* https://webbook.nist.gov/cgi/cbook.cgi?ID=C7782447 (accessed 2025-08-12).

(12) Knudsen, J.; Martin, N. M.; Grånäs, E.; Blomberg, S.; Gustafson, J.; Andersen, J. N.; Lundgren, E.; Klacar, S.; Hellman, A.; Grönbeck, H. Carbonate Formation on p(X4x)-O/Ag(111). *Phys. Rev. B* **2011**, *84* (11), 115430. https://doi.org/10.1103/PhysRevB.84.115430.

(13) Isegawa, K.; Ueda, K.; Hiwasa, S.; Amemiya, K.; Mase, K.; Kondoh, H. Formation of Carbonate on Ag(111) under Exposure to Ethylene and Oxygen Gases Evidenced by Near

Ambient Pressure XPS and NEXAFS. *Chem. Lett.* **2019**, *48* (2), 159–162. https://doi.org/10.1246/cl.180891.

(14) Turano, M. E.; Farber, R. G.; Oskorep, E. C. N.; Rosenberg, R. A.; Killelea, D. R. Characterization of Oxygenaceous Species Formed by Exposure of Ag(111) to Atomic Oxygen. *J. Phys. Chem. C* **2020**, *124* (2), 1382–1389. https://doi.org/10.1021/acs.jpcc.9b09131.

(15) Michaelides, A.; Reuter, K.; Scheffler, M. When Seeing Is Not Believing: Oxygen on Ag(111), a Simple Adsorption System? *J. Vac. Sci. Technol. A* **2005**, *23* (6), 1487–1497. https://doi.org/10.1116/1.2049302.
